# Carbonyl Profiles of Electronic Nicotine Delivery System (ENDS) Aerosols Reflect Both the Chemical Composition and the Numbers of E-Liquid Ingredients–Focus on the *In Vitro* Toxicity of Strawberry and Vanilla Flavors

**DOI:** 10.3390/ijerph192416774

**Published:** 2022-12-14

**Authors:** Alexandra Noël, Arpita Ghosh

**Affiliations:** Department of Comparative Biomedical Sciences, School of Veterinary Medicine, Louisiana State University, Baton Rouge, LA 70803, USA

**Keywords:** electronic nicotine delivery system (ENDS), electronic-cigarette, chemical profile of ENDS aerosols, *in vitro* toxicity, air-liquid interface

## Abstract

Propylene glycol (PG) and glycerin (G) are the most widely used humectants in electronic nicotine delivery system (ENDS) devices. Carbonyls are present in aerosols produced when ENDS devices heat PG and G. Whether aerosolized PG and G are innocuous to the lungs has not been established. Here, we determined the chemical profiles of ENDS aerosols containing three humectant ratios (30/70, 50/50 and 70/30, PG/VG), for three flavors (strawberry, vanilla and Catalan cream) containing either 12 or 18 mg/mL of nicotine. Additionally, we examined the *in vitro* toxicity of the strawberry- and vanilla-flavored ENDS aerosol in human lung epithelial cells (BEAS-2B) exposed at the air-liquid interface for 1 h. For strawberry- and vanilla-flavored aerosols produced by a 3rd-generation ENDS device with the same PG/G ratio, the e-liquid nicotine content of 12 and 18 mg/mL did not transfer to the aerosol with substantial differences in concentrations. Our data also indicate the presence of carbonyls in all three flavored e-cig aerosols analyzed, with levels exceeding 1 µg/puff for acetone, butyraldehyde, and acetaldehyde, in strawberry-, vanilla, and Catalan cream-flavored e-cig aerosols, respectively. Furthermore, closed-system ENDS of the fourth generation emitted trace levels of carbonyls in the aerosols (<0.3 µg/puff), while open-system tank-style ENDS of the third generation produced elevated levels of harmful chemicals, including acrolein (>1 µg/puff), formaldehyde (>5 µg/puff), and m- & p-tolualdehyde (>4 µg/puff). Moreover, under non-cytotoxic conditions, BEAS-2B cells exposed to strawberry-flavored aerosols exhibited significantly increased reactive oxygen and nitric oxide species (ROS/NOS) levels in cell media compared to air controls, while vanilla-flavored ENDS aerosols up-regulated the expression of pro-inflammatory and oxidative stress markers. Our data suggest (a) that ENDS aerosol chemical composition will vary based upon the presence and concentration of the initial e-liquid ingredients, with a pronounced impact of the flavoring components; and (b) short-term exposures to flavored ENDS aerosols may impair lung cells’ redox signaling in a flavor-specific manner.

## 1. Introduction

Over the past decade, electronic nicotine delivery systems (ENDS) have undergone a rapid innovative progression, which has led to an increase in the prevalence of vaping in the United States. According to the Centers for Disease Control and Prevention, the prevalence of ENDS use among pre-college students is ~2 million middle and high school students [1]. This is in addition to the 8 million adult ENDS users [2]. Thus, from sub-ohm through high-power vaping, and now to ‘JUULing’, since 2007, the ENDS landscape has evolved to satisfy users’ recreational and smoking aid cessation needs. With an estimated value of $6.1 billion in the United States for 2020, the ENDS market is growing exponentially [3]. This includes the sale of tank-style electronic-cigarettes (e-cigs) and pod/mod style devices (e.g., JUUL, Puff Bar, Posh Vape, Vuse, and Hyde), which are either re-usable or disposable fourth generation ENDS. Little, however, is known regarding the health effects induced by chronic inhalation of ENDS aerosols produced by third or fourth generations ENDS devices. These aerosols are composed of heated nicotine, either in the freebase or salt forms, flavoring chemicals, and humectants, including propylene glycol (PG) and glycerol (G) [4]. The rapid, recent increase in ENDS use is a major two-pronged public health concern, with alarming numbers of users among adults and vulnerable populations, including youth, and inadequate information on long-term pulmonary responses to ENDS aerosol exposures.

While third-generation ENDS devices are button-activated open systems with user-adjustable operational conditions plus user selected e-liquid, fourth-generation ENDS devices are airflow-activated closed systems with no modifiable settings and include self-contained e-liquids or exchangeable e-liquid pods. Thus, there are several ENDS-related factors that affect the vaping experience; most importantly, these are also key factors impacting the chemical profile and the toxicity of the ENDS aerosol produced. These factors include: (1) the ENDS device model. ENDS evolution from the first to the fourth generations has resulted in the sale of more than 2800 distinct types of ENDS devices that can operate with or without user-selectable settings [5]. 

(2) The ENDS liquid (e-liquid) nicotine levels, available in at least 6 freebase nicotine strengths (3 to 36 mg/mL) and several nicotine salt concentrations (up to 50 mg/mL). While 12 mg/mL is the most popular freebase nicotine concentration used by American adolescents [6], 18 mg/mL is the concentration preferred by adults [7]. In contrast, the e-liquid in fourth generation ENDS contain up to 50 mg/mL of nicotine salt, equivalent to the nicotine content found in one pack of cigarettes [8]. Noteworthy, fourth generation ENDS are currently leading the ENDS sale market [8]. 

(3) The e-liquid humectant composition. The various ratios of PG/G have a direct impact on the physicochemical characteristics of the aerosols produced [9]. PG and G are “generally regarded as safe” (GRAS) ingredients in food; however, their pulmonary safety following long-term inhalation exposures has not been thoroughly evaluated. Once heated and aerosolized by an ENDS device, the chemical interactions of PG and G produce emissions of carbonyls, including formaldehyde, acetaldehyde, and acrolein, which are known to be harmful to human health [10,11]. Concentrations of both PG or G in e-liquids range from 0 to 100%, prompting numerous possible combinations. G aerosolization through an ENDS device generates acrolein and a dense aerosol, i.e., a thick and dense cloud [11,12]. G-rich (>70%) e-liquids are associated with fruit, cream and nut flavors, and are used by a growing subclass of e-cig users, ‘the cloud chasers’, popular among youth and young adults [5]. In contrast, PG aerosolization yields higher levels of carbonyls, known to be mouth and throat irritants, and thus confer the ‘throat hit’ sensation for both flavor and nicotine, while generating a thinner, more discreet, aerosol [5,11,12]. PG-rich (>70%) e-liquids are mostly associated with tobacco, menthol and beverage flavors [5]. Further, these humectants have been shown to cause toxicity *in vitro* [13]. E-cig aerosols composed of 50/50 PG/G or 100% G, reduced the metabolic activity of human bronchial epithelial cells exposed at the air-liquid interface, while 100% PG e-cig aerosols increased the release of cytokines, including IL-10 and CXCL1 [13].

(4) The e-liquid flavors, representing over 16,000 distinct products, can be grouped into several categories, including: fruits, beverages, sweets, nuts, cream, menthol, seasonings and tobacco [5,14]. Fruit and fruit beverage, dessert, as well as candy and sweet, are part of the United States most trendy e-liquid flavor categories [15,16]. 

Taken together, there are multiple factors, including ENDS device settings, e-liquid composition, and user topography, that can affect the vaping experience. Flavors and cloud production are selected subjectively by ENDS users [15], impact the physicochemical profile of the aerosol produced, and thus, ultimately the pulmonary toxicity.

For exposures that occur via the inhalation route, the bronchial epithelium, whose composition includes basal cells, club cells, goblet cells, and ciliated cells, is at the intersection of host-external environment interactions [17,18]. Due to their strategic location in the airways, bronchial epithelial cells play many key roles in the maintenance of pulmonary homeostasis, including the ability to elicit innate and adaptive immune responses, as part of the lungs first line of defense against inhaled pathogens and pollutants; the capacity to produce cytokines, implicated in pro- and anti-inflammatory reactions; its function as a physical barrier; and in the removal of xenobiotics through the mucociliary clearance [17,18,19]. Thus, bronchial epithelial cells are vital ‘players’ involved in lung structure and function, and their dysfunction is associated with the pathogenesis of several lung diseases, including asthma and emphysema [17,18]. Thus far, evidence has shown that e-liquid ingredients (PG, G, flavoring chemicals, nicotine) following heating and aerosolization through an ENDS device can be toxic to lung cells [13,20,21]. The long-term pulmonary effects of ENDS, however, remain largely unknown.

Overall, the industry of new alternative tobacco-related products is changing rapidly and scientific evidence supporting the ‘safe use of ENDS’, is scarce. Together, (1) the increasing popularity of ENDS use among youth and young adults, (2) the broad and diverse scope of ENDS products, which challenges toxicity and regulatory assessments, and (3) the paucity of data on inhaled ENDS aerosols and pulmonary responses in presumed healthy but still vulnerable populations, emphasize the critical need to determine health effects of long-term exposures to these inhalable aerosols, to provide scientific evidence for future regulations on new alternative tobacco products. Thus, the overarching objectives of this study were (1) to determine the chemical profiles of ENDS aerosols containing three popular humectant ratios (PG/G: 30/70, 50/50, 70/30), flavors (vanilla, strawberry and Catalan cream) and nicotine forms (freebase or salt) and (2) to evaluate the *in vitro* toxicity in human bronchial epithelial cells exposed to strawberry- and vanilla-flavored ENDS aerosol at the air-liquid-interface (ALI).

## 2. Methods

### 2.1. ENDS Aerosol Generation and Chemical Characterization

All e-liquids were purchased online from EC Blend (Medford, OR). Fourth-generation ENDS (JUUL, Puff Bar and Posh Plus) were purchased from either JUUL Labs (San Francisco, CA, USA) or from InLine Vape LLC (Puff Bar and Posh Plus). Each e-liquid ingredient (PG/G, flavor and nicotine concentration) was tested for its most popular variables (ratios of PG/G: 70/30; 30/70; and 50/50; strawberry, vanilla and catalan cream flavors; 12 mg/mL and 18 mg/mL free base nicotine). We produced the third-generation e-cig aerosols with a Joyetech eVic VTC mini mod connected to a SCIREQ e-cigarette aerosol generator (Scireq^®^, Montreal, QC, Canada), as described in [22]. We used an atomizer resistance of 0.15 ohm combined with a battery voltage set at 4.2 V. These ENDS aerosols were not produced under ‘dry puff’ conditions. We produced the fourth-generation ENDS aerosols by connecting the ENDS devices (JUUL, Puff Bar, Posh Plus) to a peristaltic pump (Cole-Palmer Masterflex L/S), as described in [21]. All ENDS exposures were conducted following a vaping topography of 1 puff every 30 s and a 55 mL puff volume, as per the CORESTA standards [23]. We identified and quantified the nicotine, PG and G concentrations found in the ENDS aerosols by collecting the aerosol on a 44 mm Cambridge filter pad at a loading regimen of 1 L/min. Quantification was subsequently done by gas chromatography with a flame ionization detector (GC/FID) Limits of detection (LOD) were 0.0812 mg, 0.0840 mg and 0.101 mg for nicotine, PG, and G, respectively. In those ENDS aerosols we also quantified the presence of selected carbonyls. Samples for carbonyl quantification were collected in 2,4-dinitrophenylhydrazine (DNPH) tubes at a loading regimen of 1 L/min and were subsequently analyzed using the EPA method TO-11A based on high performance liquid chromatography (HPLC). Minimum detection limits (MDL) were 0.0226 µg for acetaldehyde, acetone and formaldehyde, 0.0225 µg for acrolein, benzaldehyde and butyraldehyde, and 0.0452 µg for m- & p-tolualdehyde. Further, for the aerosols produced by the fourth-generation devices, we also quantified the concentration of selected organic acids. Following sample collection, the analysis for organic acids was conducted by ion chromatography. We collected all samples at the Inhalation Research Facility at Louisiana State University and shipped them overnight on dry ice to Enthalpy Analytical LLC (Durham, NC, USA) for analysis.

### 2.2. In Vitro Assay

We examined the *in vitro* toxicity of strawberry-flavored and vanilla-flavored ENDS aerosols in human lung epithelial cells (BEAS-2B) exposed at the air-liquid interface (ALI) for 1 h. These e-liquids all contained 18 mg/mL of nicotine and 30/70 PG/G content (see above). 24 h following the exposure, we evaluated cell viability, reactive oxygen species (ROS) and levels of nitric oxide (NO), as well as gene expression.

### 2.3. Cell Culture

BEAS-2B cells (human bronchial epithelial cell line; ATCC CRL-9609) were grown in T-75 tissue culture flasks and maintained in DMEM medium with 10% fetal calf serum, 100 U/mL penicillin, 100 μg/mL streptomycin, as previously described in [21]. Cells were kept in a tissue culture incubator (37 °C, 5% CO_2_). We used cells from passages 8 to 16. We seeded ~1 × 10^4^ cells on 24 mm transwell inserts (0.4-μm pore size polyester membrane insert, catalog #3450, Corning Incorporated, Corning, NY, USA) in 6-well plates. Once the cells were on the transwell inserts, we changed the cell media every 2–3 days for 21–23 days. The cells that were grown on the transwell inserts were exposed to the ENDS aerosols on day 21–23. 

### 2.4. Air-Liquid Interface (ALI) Exposures

We used a customized ALI exposure system (Vitrocell Systems, GMBH, Waldkirch, Germany) that has been previously described in [20]. In brief, this ALI exposure system is composed of a stainless-steel exposure module that can accommodate three 6 well/24 mm diameter inserts, plus one microbalance. This exposure module contains the cells grown at the ALI and is connected to a distribution system that allows for the aerosol exposures. For the control cells, there is a separate stainless steel exposure module made for three 6 well/24 mm diameter inserts. This control exposure module is connected to a medical-grade compressed air distribution system. The medical grade compressed air is also used to dilute the ENDS aerosol. The ALI exposure system is equipped with a water bath, allowing for warm water (36–37 °C) to be circulated around the exposure modules to keep the cells at 37 °C. To investigate the *in vitro* toxicity to BEAS-2B cells induced by strawberry- and vanilla-flavored ENDS aerosols, we connected the Joyetech eVic VTC mini mod (third-generation ENDS), as described above, to the distribution system of our Vitrocell ALI exposure system. Additionally, the ENDS device was operated with the settings and topography profile described above. For scientific rigor, the same experiment was performed three independent times and each flavor had its own control group. To be representative of real-world vaping scenarios, our exposure duration of 1-h or 120 puffs, is based on the topography of ENDS users, with previous reports showing a median number of 132 puffs per day [24]. Thus, we exposed BEAS-2B cells to either strawberry-flavored, vanilla-flavored ENDS aerosols or medical grade compressed air for 1 h for 1 day. Following the exposure, we returned the cells to the incubator for 24 h, at which point biological endpoints were measured.

### 2.5. Cell Viability 

We evaluated cell viability 24 h after the ENDS aerosol exposure via the trypan-blue dye-exclusion assay. We assessed the viable cell counts by adding a 10 μL aliquot of cell suspension into a TC10 counting slide (catalog #1450015, Bio-Rad Laboratories, Hercules, CA, USA), which was inserted in a TC20 automated cell counter (catalog #1450102, Bio-Rad Laboratories, Hercules, CA, USA). We evaluated all samples in duplicate.

### 2.6. Extracellular Reactive Oxygen Species (ROS) Measurements

We used the OxyBURST Green assay (dihydro-2′,4,5,6,7,7′-hexafluorofluorescein-BSA (H2HFF), Catalog # D2935, Invitrogen, Thermo Fisher Scientific, Waltham, MA, USA) to evaluate the extracellular ROS production in the cell media collected from the basal side of the Transwell. As described in [20], we followed the manufacturer’s instructions. We used a Tecan Infinite 2000 spectrophotometric plate reader (Tecan Group Ltd., Mannedorf, Switzerland) to measure fluorescence at a 497 nm excitation and 527 nm emission wavelengths. We normalized the cell media fluorescence based on the cell count for each sample. Comparisons between groups were made with the fluorescence values for the air control groups set at 100%. We evaluated all samples in triplicate.

### 2.7. Extracellular Nitric Oxide (NO) Measurements

We used the Griess reagent assay (catalog #30,100, Biotum, Fremont, CA, USA) to evaluate the extracellular NO production in the cell media collected from the basal side of the Transwell. As described in [20], we followed the manufacturer’s instructions. We used a Tecan Infinite 2000 spectrophotometric plate reader (Tecan Group Ltd., Mannedorf, Switzerland) at a wavelength of 490 nm to evaluate the absorbance. We normalized the cell media absorbance based on the cell count for each sample. Comparisons between groups were made with the absorbance values for the air control groups set at 100%. We evaluated all samples in triplicate.

### 2.8. RNA Extraction and qRT-PCR

Twenty-four hours post-exposure, we collected the BEAS-2B cells, which were pooled from the three technical replicates, for subsequent RNA extraction. As previously described [20], we conducted RNA isolation using the Qiagen RNeasy Micro Kit (catalog number 74004) with Trizol/chloroform extraction. We used a NanoDrop ND-1000 Spectrophotometer (NanoDrop, Wilmington, DE, USA) to determine the RNA concentration and purity. cDNA was prepared using the iScript cDNA Synthesis kit (Bio-Rad Laboratories, Hercules, CA, USA) and amplified with a T100 Thermal Cycler (Bio-Rad Laboratories, Hercules, CA, USA). We used either designed primers, as listed in Table 1 or inventoried TaqMan gene expression assays primers-probe sets (Applied Biosystems, Waltham, MA, USA) to conduct quantitative real-time PCR (Applied Biosystems 7300 Real Time PCR System) on cDNA samples. We used the 2−ΔΔCt method to calculate fold-changes of exposed groups compared to air controls. For normalization, we used β-ACTIN as the housekeeping gene. We expressed the results as fold-change over air controls.

### 2.9. Statistical Analyses

We performed the statistical analyses using GraphPad Prism version 9 (GraphPad Software, San Diego, CA, USA). The results are reported as mean ± standard error of the mean (SEM). Some results are reported as percent change compared to the respective air control group, which was set at 100%. We used either a Student-t test (2 groups) or a one-way analysis of variance (ANOVA) (3 groups or more) followed by a Tukey’s post hoc test, to test whether there were any statistically significant differences between groups. Results were considered statistically significant with a *p*-value < 0.05 or a fold-change > ±1.5 for the gene expression data. 

## 3. Results

### 3.1. ENDS Aerosols Composed Solely of PG and G Generated by a Tank-Style Device Contain Carbonyls

**Figure 1:**
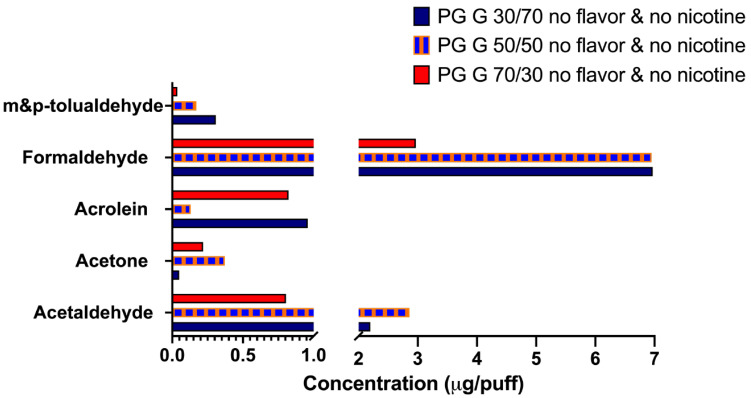
ENDS aerosols composed solely of PG and G contain carbonyls. Carbonyls levels found in ENDS aerosols composed solely of PG and G (with no added flavor or nicotine). On average 40 puffs were collected. This chemical profile screening was comprised of a one-time carbonyls analysis of the various ENDS aerosol samples.

To determine whether the humectants in the e-liquid contribute substantially to the emission of carbonyls in the ENDS aerosols, we conducted the chemical analysis of ENDS aerosols generated from e-liquids composed solely of PG and G (without any nicotine and flavoring chemicals). We found that ENDS aerosols composed solely of the humectants, PG and G in ratios of 30/70, 50/50, and 70/30, with no nicotine and no flavor, produced elevated levels of carbonyls (Figure 1). We observed that the ENDS aerosols generated from the 30/70 and 50/50 PG/G e-liquids yielded high concentration of formaldehyde (>6 µg/puff) and of acetaldehyde (>2 µg/puff) (Figure 1). These data also indicated that higher carbonyls concentrations were measured in G-rich ENDS aerosols (>50%). This baseline level of carbonyls is mainly due to the thermal degradation of PG and G following heating and aerosolization through an ENDS device [25].

### 3.2. Carbonyls Are Present in Strawberry-, Vanilla- and Catalan Cream-Flavored E-Cig Aerosols

**Figure 2:**
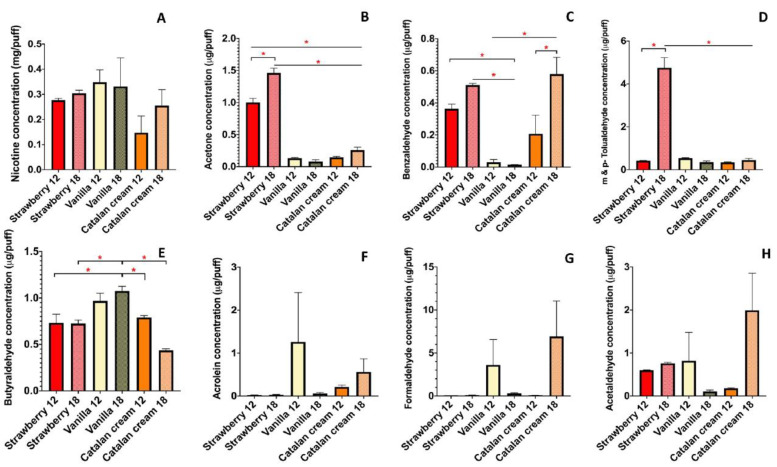
Carbonyls are present in strawberry, vanilla and Catalan cream flavored e-cig aerosols. (**A**–**H**) E-cig aerosol concentrations for nicotine, acetone, benzaldehyde, m & p-tolualdehyde, butyraldehyde, acrolein, formaldehyde, and acetaldehyde. N = 3 per condition. ANOVA followed by a Tukey post hoc test. * *p* < 0.05. On average 40 puffs were collected.

For strawberry- and vanilla-flavored e-liquids containing 12 and 18 mg/mL of nicotine, we observed that the e-liquid nicotine content does not transfer to the e-cig aerosol with substantial differences in concentrations (Figure 2A). Albeit not statistically significant, a more notable difference in aerosol nicotine content for the 12 and 18 mg/mL concentrations was observed for the Catalan cream-flavored e-liquid (Figure 2A). Overall, these data show that a 6 mg/mL e-liquid nicotine concentration difference may not be sufficient to translate into significant e-cig aerosol nicotine concentration differences, and that efficient e-liquid nicotine aerosol transfer may be flavor-specific, and thus dependent on the interactions between the humectants and the flavoring chemicals (Figure 2A).

In addition, for those three flavored e-cig aerosols, we determined the concentrations of several carbonyls, including acetaldehyde, acetone, acrolein, benzaldehyde, butyraldehyde, formaldehyde, and m & p- tolualdehyde (Figure 2B–H). We found that strawberry-flavored e-cig aerosols contained high levels of acetone (>1 µg/puff). In the case of the 18 mg/mL nicotine samples, m & p- tolualdehyde levels were >4.7 µg/puff (Figure 2B,D). For the vanilla-flavored e-cig aerosols, we found high levels of butyraldehyde (~1 µg/puff), while for the 12 mg/mL nicotine samples, we found elevated levels of acrolein (>1 µg/puff) and formaldehyde (>3.6 µg/puff) (Figure 2E–G). For the Catalan cream ENDS aerosols, with the 18 mg/mL nicotine samples, we found non-significantly elevated levels of acetaldehyde (>1.9 µg/puff) and formaldehyde (>6.9 µg/puff) (Figure 2G,H). Nonetheless, the chemical profiles of these Catalan cream e-cig aerosols show that they contain harmful chemicals. Taken together, these results show that carbonyl emissions from third-generation ENDS devices may be flavor-specific and suggest that some flavors may produce a chemical profile that could be more harmful than others.

### 3.3. ENDS Aerosols Produced by Fourth Generation Devices Contain Trace Levels of Carbonyls but Generate High Levels of Benzoic Acid

**Figure 3:**
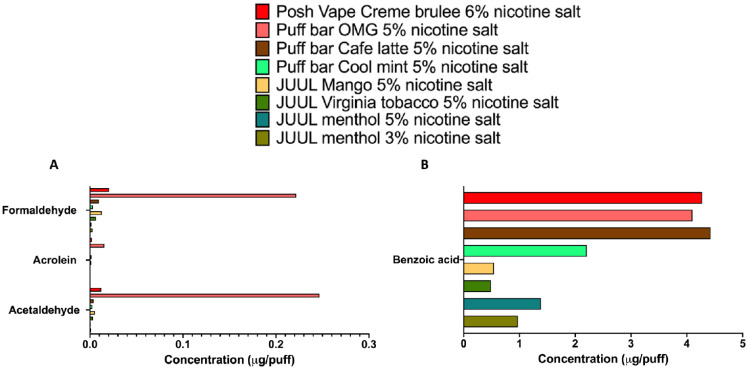
Fourth-generation ENDS aerosols contain trace levels of carbonyls (**A**), while Puff Bar and Posh Vape devices generate high levels of benzoic acid (**B**). On average 40 puffs were collected. This chemical profile screening was comprised of a one-time carbonyls and organic acids analysis of the various ENDS aerosol samples.

The results in Table 2 show that the aerosol nicotine concentration measured for JUUL devices containing 5% nicotine salt varied based on flavor (0.09 to 0.145 mg/puff), with the JUUL mango-flavored aerosol containing the highest nicotine levels. As for the other fourth-generation ENDS devices evaluated, including Puff Bar and Posh Vape devices, the aerosol nicotine concentrations were higher than those found for JUUL, although the labeled nicotine salt content for those devices were similar to JUUL (either 5 or 6%) (Table 2). The Puff Bar Cool mint-flavored aerosol showed the highest nicotine concentration, with 0.378 mg/puff (Table 2). It was previously reported that 30/70 is the PG/G content used in the e-liquids for JUUL and other fourth-generation devices [26]. In the JUUL aerosols, we found that the PG to G ratio was ~3 for all the three flavors (menthol: 1.55/0.515 = 3.01; Virginia tobacco: 1.66/0.544 = 3.05; and mango: 2.09/0.639 = 3.27), while for the disposable ENDS devices (Puff Bar and Posh Vape), the PG to G ratio ranged between 1.2–1.5 (Puff Bar Cool mint: 4.05/3.26 = 1.24; Puff Bar Café latte: 1.45/1.19 = 1.22; Puff Bar Orange-mango-guava (OMG): 3.76/2.56 = 1.47; and Posh Vape Crème brûlée: 4.58/3.06 = 1.49) (Table 2). In addition, for the ENDS aerosols produced by JUUL, Puff Bar, and Posh Vape devices, we found only trace levels of carbonyls (<0.3 µg/puff) (Figure 3A), while the Puff Bar and Posh Vape devices yielded aerosols with high levels of benzoic acid (>2 µg/puff) (Figure 3B). Overall, our data demonstrate that ENDS devices within the same closed system category can produce ENDS aerosols with distinct chemical profiles.

### 3.4. 1-h of Exposure at the ALI to Strawberry-Flavored E-Cig Aerosols Cause Oxidative Stress, While Vanilla-Flavored e-Cig Aerosols Induce Transcriptional Changes in BEAS-2B Cells

**Figure 4:**
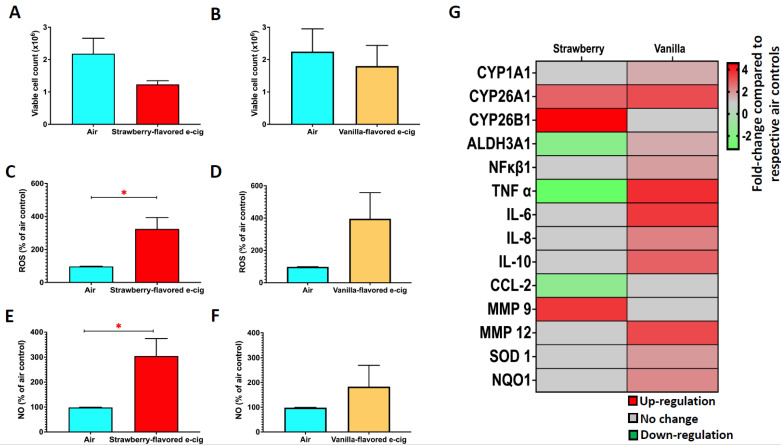
1-h of exposure at the ALI to strawberry-flavored e-cig aerosols cause oxidative stress, while vanilla-flavored e-cig aerosols induce transcriptional changes in BEAS-2B cells. (**A**,**B**) 1-h exposure to strawberry- and vanilla-flavored ENDS aerosols at the ALI is not cytotoxic to BEAS-2B cells. (**C**–**F**) Strawberry-flavored aerosols induced significantly increased reactive oxygen and nitric oxide species (ROS/NO) levels in cell media. (**G**) Vanilla-flavored ENDS aerosols dysregulated more genes than strawberry-flavored ENDS aerosols in BEAS-2B cells. Significance > ±1.5-fold change when compared to respective air controls. For each biological outcome, N = 3 independent experiments, each conducted in technical triplicates. Student *t*-test. * *p* < 0.05.

Following the assessment of the chemical profiles of the three e-cig aerosols (Figure 2), we examined the *in vitro* toxicity of the top two flavors that produced the most significant carbonyl emissions in e-cig aerosols when present with 18 mg/mL of nicotine. We exposed human lung epithelial cells (BEAS-2B) at the ALI for 1 h to strawberry-flavored and vanilla-flavored e-cig aerosols. The e-liquids all contained 18 mg/mL of nicotine and a PG/G ratio of 30/70. Twenty-four hours post-exposure, we found that a 1-h exposure to strawberry- and vanilla-flavored e-cig aerosols at the ALI was not cytotoxic to BEAS-2B cells (Figure 4A,B). Thus, this *in vitro* exposure scenario allows for obtaining data under non-cytotoxic conditions. Despite not being toxic to BEAS-2B cells, strawberry-flavored e-cig aerosols significantly increased reactive oxygen and nitric oxide species (ROS/NO) levels in the cell media, while exposures to vanilla-flavored e-cig aerosols had no significant effect on ROS/NO levels (Figure 4C–F). These results suggest that strawberry-flavored aerosol may impair lung cells’ redox signaling. Although increases in ROS & NO levels were induced by strawberry-flavored ENDS aerosols, at the molecular level, we found that vanilla-flavored ENDS aerosols dysregulated more genes (11 genes) than strawberry-flavored (6 genes) ENDS aerosols in BEAS-2B cells (Figure 4G). This shows that 1-h of exposure to vanilla-flavored ENDS aerosols is sufficient to increase at the transcriptomic level biotransformation (*CYP26A1*), pro-inflammatory (*TNF-α*, *IL-6*, *IL-8*) and oxidative stress (*NQO1*) markers in BEAS-2B cells. Overall, these data indicate that the type of *in vitro* toxicity, with specific effects observed at the cellular or molecular levels, induced by e-cig aerosols in human bronchial epithelial cells, may be flavor-specific.

## 4. Discussion

We determined the chemical profiles of e-cig aerosols containing the three most popular humectant ratios (PG/G: 30/70, 50/50 and 70/30), one flavor from each of the three top flavor categories (fruit: strawberry; desserts: vanilla; and candy flavors/sweet (non-fruity): Catalan cream), as well as two preferred freebase nicotine concentrations: 12 and 18 mg/mL [6,7] (Figure 1 and Figure 2). Our results demonstrate that third generation ENDS aerosols composed solely of humectants (PG and G), can contain elevated levels of acetaldehyde and formaldehyde (>2 µg/puff) (Figure 1). Further, our data indicate the presence of carbonyls in all three flavored e-cig aerosols analyzed, with levels exceeding 1 µg/puff for acetone, butyraldehyde, and acetaldehyde, in strawberry-, vanilla, and Catalan cream-flavored e-cig aerosols, respectively (Figure 2). One notable difference between ENDS aerosols generated by third and fourth generation devices was in the carbonyl concentrations emitted by those ENDS (Figure 2 and Figure 3). Closed-system fourth generation ENDS emitted trace levels of carbonyls in the aerosols (<0.3 µg/puff) (Figure 3), while open-system tank-style third generation ENDS produced elevated levels of harmful chemicals, including acrolein (>1 µg/puff), formaldehyde (>5 µg/puff), and m- & p-tolualdehyde (>4 µg/puff) (Figure 3). Similar trends have been previously published where levels of carbonyls emitted from fourth-generation devices are much lower than those emitted from third-generation devices [27]. Thus, our data show that e-cig aerosol chemical composition is complex and can vary based upon the presence and concentration of the initial e-liquid ingredients. Additionally, our *in vitro* data suggest that short-term (1 h) exposures (a) to strawberry-flavored ENDS aerosols may impair lung cells’ redox signaling, and (b) to vanilla-flavored ENDS aerosols, may up-regulate the expression of pro-inflammatory and oxidative stress markers, all without eliciting any significant cytotoxicity in BEAS-2B cells (Figure 4). These differential toxicity profiles may be due to the presence of flavoring chemicals as well as carbonyls in the ENDS aerosols. Indeed, the strawberry-flavored 18 mg/mL of nicotine ENDS aerosols contained high levels of acetone and m- & p-tolualdehyde (>1 µg/puff), while the vanilla-flavored 18 mg/mL of nicotine aerosols contained elevated concentrations of butyraldehyde (>1 µg/puff) (Figure 2). This suggests that ENDS aerosols *in vitro* toxicity may be flavor- and chemical profile-specific. In a public health context, our results demonstrate that e-cig aerosols composed solely of humectants or including nicotine and flavorings, contain acetaldehyde, acetone, acrolein, and formaldehyde (Figure 1, Figure 2 and Figure 3), which are respiratory toxicants enumerated on the harmful and potentially harmful chemicals list established by the FDA for tobacco products [28]. Thus, suggesting that following prolonged exposures inhaled e-cig aerosols could potentially harm lung cells.

The main constituents of e-liquids include the base humectants, PG and G, which by weight will constitute ~90 to 97% of the e-liquid [25]. Flavoring chemicals can occupy 1 to 4% (10 to 40 mg/mL) of the e-liquid volume, while freebase nicotine levels usually range from 3 to 3.6% (3 to 36 mg/mL) [29]. The thermal degradation or the pyrolysis and oxidation of the base humectants PG and G when aerosolized through an ENDS device are known to produce aldehyde by-products [25]. This can explain the levels of m- & p-tolualdehyde, formaldehyde and acetaldehyde detected in our e-cig aerosols composed solely of humectants (Figure 1). Evidently, the presence and concentrations of these aldehydes will depend on the e-liquid composition and the e-cig device operational settings and thus, the exposure conditions [25]. Comparably to our results, several other studies have reported detectable levels of acetaldehyde, acrolein, butyraldehyde, and formaldehyde in flavored and unflavored e-cig aerosols [12,25,30,31,32,33]. In one study, acetaldehyde was the most prevalent aldehyde found in 26 e-cig aerosols analyzed (median levels: 1520 µg/m^3^), while formaldehyde was detected in 92% of samples (median levels: 626 µg/m^3^), with levels exceeding occupational exposure limits [25]. Additionally, it was previously demonstrated that compared to unflavored e-cig aerosols containing solely PG/G plus nicotine, flavored e-cig aerosols with similar composition but with added flavoring chemicals (e.g., café mocha, midnight apple), contained higher levels of acetaldehyde and lower levels of acrolein [34]. These data indicate that the addition of flavoring chemicals to the e-liquid can either increase or decrease the formation of aldehydes during the heating and aerosolization process of using an ENDS device. This supports our data where we similarly showed that e-cig aerosols containing solely humectants could generate detectable levels of aldehydes (Figure 1) and that adding nicotine and flavoring chemicals impacted those levels in a flavor-specific manner (Figure 2). Importantly, the transfer of the flavoring chemicals from the e-liquid to the e-cig aerosol has been shown to be e-cig device setting-dependent, meaning that the atomizer coil, voltage, and wattage applied to the ENDS device will influence the composition and concentration of the compounds transferred to the aerosol, with very efficient transfers (~98%) observed when using voltages of 3 or 5V [13,35]. This is similar to the voltage we used in our study. Taken together, our data along with other reports [12,25,30,31,32,33,34] indicate that multiple factors influence the generation of aerosolized aldehydes in e-liquids and that the source of the aerosolized aldehydes is not solely due to the presence of flavoring chemicals in e-liquids. Further, it is important to note that PG and G from different e-liquid brands can produce different levels of carbonyls. In addition, batch-to-batch differences from a same product have previously been reported [36]. Thus, the results presented herein may be brand-specific rather than solely e-liquid type-specific, and therefore may not be generalizable to all e-liquids.

We found that carbonyl levels emitted from fourth-generation ENDS (JUUL, Puff Bar and Posh Plus) were much lower than those emitted from third-generation e-cig devices (<0.3 µg/puff versus > 5 µg/puff, respectively) (Figure 2 and Figure 3), with similar trends also reported by others [27]. Although one may conclude that these data support that the use of fourth generation ENDS as being ‘safer’ for human health compared to the use of tank-style ENDS devices, thus far, human and experimental evidence show differential responses following exposures to these distinct types of ENDS devices. It was recently shown it a study using sputum of smokers, third-generation e-cig users, fourth-generation e-cig users, and non-smokers (controls), that the use of fourth-generation ENDS, containing high nicotine salt levels (35 to 59 mg/mL), induced a distinct suppressive immune disruptive pattern in the lungs compared to the use of ENDS from previous generations and even from cigarettes [4]. Thus, it was proposed that nicotine salt drove the immunosuppressive responses in the lungs [4]. This contrasts with the presence of high aldehyde levels found in third-generation ENDS aerosols, which are known to affect cytotoxicity, inflammation and oxidative stress pathways in lung cells [13,37]. Moreover, the data from Table 2 and Figure 3 show that although JUUL and disposable Puff Bar and Posh Vape devices are closed ENDS (with no modifiable operational settings), that feature similar nicotine salt concentrations (5 or 6%), the ENDS aerosols generated by these devices contain different levels of the primary e-liquid constituents (Table 2). This suggests that the operational settings, including wattage, resistance and voltage, applied to these devices from the same fourth generation may be different and that the chemical profiles of those closed ENDS aerosols may be notably distinctive. Overall, while open (third generation devices) versus closed (fourth generation devices) ENDS have distinct operational settings, which influence the chemical profile of the aerosols generated [9], the presence of harmful and potentially harmful chemicals as well as the form of nicotine (freebase versus salt) in the ENDS aerosols may be key factors to consider when evaluating ENDS toxicity.

It is also important, however, to bear in mind that in real-world exposure scenarios, when inhaled, ENDS aerosols will be subjected to deposition along the three regions of the respiratory tract (naso-pharyngeal, trachea-bronchial, and alveolar) [38]. The physical properties of the ENDS aerosol will be influenced by multiple factors, including e-liquid composition, ENDS device settings, and vaping topography. Aerosol density and size distribution will mainly determine pulmonary deposition along the respiratory tract, in addition to the aerosol also being partly exhaled [38,39]. Thus, the pulmonary absorption of the carbonyls found in the ENDS aerosols will be influenced, among others, by the chemical properties of the specific carbonyl, for instance, acrolein, acetaldehyde, and formaldehyde are primarily upper respiratory tract irritants [40]; the lung morphology, the lung disease state, the breathing pattern, as well as the ENDS aerosol particulate phase [38,39]. Whether the adsorption of carbonyls onto the ENDS aerosol particulate phase increases the distribution of these compounds in the lower respiratory tract (bronchial and alveolar regions) is currently unknow, and thus merit future investigation. Nonetheless, several of these carbonyls have permissible or recommended exposure limits (PEL/REL) in occupational settings in the Unites States. Acetone has a time-weighted average (TWA) REL of 250 PPM or 590 mg/m^3^; acrolein a TWA PEL of 0.1 PPM or 0.25 mg/m^3^; acetaldehyde a TWA PEL of 200 PPM or 360 mg/m^3^; and formaldehyde a TWA PEL of 0.75 PPM or 0.92 mg/m^3^[41]. Although benzaldehyde, m- & p-tolualdehyde, butyraldehyde, and benzoic acid do not have established PEL or REL, this does not mean these carbonyls are harmless. Globally, this highlights that more research is needed to ensure that ENDS users’ daily inhaled concentration of carbonyls, particularly acrolein and formaldehyde, which have low PEL, do not exceed recommended levels when using third-generation devices.

On average, there are 2 to 10 different flavoring chemicals per bottle of flavored e-liquid [42]. Thus, e-liquids that include flavoring chemicals are complexes mixtures that result in the generation of a complex ENDS aerosol composed of the parent compounds (PG, G, nicotine and flavorings) plus the thermal degradation by-products of those parent compounds (e.g., aldehydes). Several studies have showed that flavoring chemicals have the capacity to alter the bioavailability and cytotoxicity of nicotine and/or the humectants of the e-liquid, upon heating and aerosolization into the ENDS device [13,37]. Further, e-liquid components can interact and result in the formation of new compounds in aged e-liquids, e.g., flavor aldehyde PG/G-acetals [43,44]. This includes the documented emission of vanillin PG-acetals and benzaldehyde PG-acetals in vanilla-flavored and berry/fruit-flavored ENDS aerosols, respectively [43]. Benzaldehyde, an aromatic aldehyde, is a main flavoring chemical used to produce fruit flavors [45], in addition, flavoring chemicals listed in strawberry-flavored e-liquid include, methyl cinnamate, ethyl maltol, ethyl butyrate, ethyl acetate, isoamyl acetate and benzyl alcohol [46,47]. Levels of benzaldehyde, a known respiratory tract irritant, ranging from 0.17 to 4.71 µg/puff were detected in cherry-flavored e-cig aerosols, while levels varying 0.0008 and 0.34 µg/puff were reported in non-cherry flavored e-cig aerosols [25,45,48]. This is in line with our study, where for the three non-cherry flavors analyzed, benzaldehyde was present at concentration below 0.6 µg/puff, with strawberry and Catalan cream flavors showing the highest levels (Figure 2). The flavoring chemicals used to create vanilla flavors include vanillin and ethyl vanillin; both aldehydes [47]. Vanillin ranks second among the most used flavoring chemicals in e-liquids [44]. With a presence in 42% of 320 e-liquids tested, vanillin was the most frequently detected flavoring chemical among all e-liquid flavor categories combined [42]. When refining the analysis to ‘dessert’ only e-liquid flavor category, the presence of vanillin flavoring was detected in 82% of the e-liquids [42]. While the concentration of vanillin ranged from 0.4 to 13.5 mg/mL in e-liquids, vanillin and ethyl vanilla have been shown to be respiratory irritants and have inflammatory capacities [29,42,48]. Overall, it has been shown that heating flavoring chemicals through e-cig devices can led to the emission and subsequently the inhalation of flavoring chemical plus their degradation by-products by e-cig users [49]. This clearly shows that e-cig aerosols are complex mixtures, with the presence of additional degradation by-products that are formed during the heating process [49].

Thus, e-liquids containing several flavoring chemicals result in more complex ENDS aerosols, due to the interactions between the ingredients and the formation of new emitted compounds, in contrast to ENDS aerosols generated from an e-liquid containing a single or fewer (1 to 3) flavoring chemicals [50]. In our study, strawberry-flavored e-cig aerosol, which, as described above is a more complex mixture than vanilla-flavored e-cig aerosol, induced oxidative stress responses, while such effects were not significantly different in the vanilla exposed cells (Figure 4). However, at the molecular level, vanilla-flavored e-cig aerosol dysregulated more genes than the strawberry-flavored e-cig aerosol (Figure 4), with vanillin, a flavoring aldehyde, documented to induce molecular damage, including DNA damage responses [51]. Oxidative stress can be observed when the cell produces ROS, including superoxide anion and hydroxyl radical, in excessive quantities [52]. E-cig aerosols are known for producing ROS, since nicotine, as well as some flavoring chemicals, are recognized pro-oxidant compounds [52,53,54], and e-cig aerosols can generate 7 × 10^11^ free radicals per puff [55]. E-cig aerosol-induced oxidative stress responses have previously been reported in *in vitro* models, with increase production of hydrogen peroxide (H_2_O_2_) equivalent [54], as well as in in vivo models, with decrease levels of total glutathione in BALF of exposed mice [54]. Other *in vitro* studies, albeit not conducted at the ALI with heated and aerosolized e-cig aerosols, showed that human lung epithelial cells as well as fibroblasts exposed to flavoring chemicals used in e-liquids led to increased levels of ROS [48,51]. Thus, the increased extracellular ROS levels we observed following the exposure of BEAS-2B cells to strawberry-flavored e-cig aerosol (Figure 4) are in line previous reports [48,51,54]. Increased ROS production can result in dysregulated cellular redox signaling [54]. Further, it was previously reported that strawberry-flavored ENDS aerosol was most toxic to H292 cells exposed at the ALI, compared to tobacco-, piña colada-, menthol- and coffee-flavored ENDS aerosols [13]. This toxicity was evidenced by significantly decreased cellular viability and metabolic activity, as well as increased levels of IL-10, CXCL1, CXCL2, and CXCL10 in the media [13]. These results highlight that strawberry flavor is more cytotoxic than many other flavored e-liquids. Likewise, increased *in vitro* toxicity of strawberry-flavored e-liquids have been demonstrated in several studies using different cell lines, including CALU3 airway epithelial cells [13,56], pulmonary artery smooth muscle cells [57], and retinal pigment epithelial cells [58]. Our data show that this is also the case for BEAS-2B cells exposed at the ALI to heated and aerosolized strawberry-flavored e-cig aerosols generated by a third-generation device (Figure 4). Furthermore, our short-term exposure data correlate with effects seen in e-cig users since short-term inhalation of ENDS aerosols increased oxidative stress levels in the serum of ENDS users naïve to smoking [59]. Moreover, in a crossover study, the serum levels of soluble NOX2 and of 8-isoprostane, which are markers of reactive oxidants and oxidative stress, respectively, were increased 30 min after inhaling 9 puffs of e-cig aerosol when compared to levels measured before the vaping session [52]. Overall, our data (Figure 4) along with other experimental [13,56,57,58] and epidemiological [52,59] evidence suggest that acute inhalation of e-cig aerosol can disrupt the redox state of pulmonary cells, which can translate into early adverse effects that can lead to the development of pulmonary diseases [54].

Aromatic flavor aldehydes are a class of flavoring chemicals that include vanillin [44]. Vanillin from e-liquids was showed to have the highest cytotoxic potential in a high throughput screening assay [60], and to induce oxidative stress and DNA damage pathways in cells [50,51]. In addition, vanillin PG-acetal was shown to be more toxic to BEAS-2B cells than vanillin, the parent compound [44]. Furthermore, vanillin and ethyl vanillin flavoring chemicals are monoamine oxidase inhibitors, found in e-liquids at levels that could lead to physiological inhibitory activity [61]. Thus, the properties of aldehydes, particularly vanillin, strongly suggests the possibility of adverse effects following chronic exposures to vanilla-flavored e-cig aerosols. Interestingly, we found that an acute 1-h exposure of BEAS-2B cells to vanilla-flavored e-cig aerosol at the ALI was not cytotoxic and did not significantly disrupt the redox state of the cells (Figure 4). The expression of several genes, however, were significantly dysregulated by this exposure (Figure 4). While the number of dysregulated genes by the vanilla-flavored e-cig aerosol was almost double that of the genes dysregulated by the strawberry-flavored e-cig aerosol (Figure 4), the 11 genes dysregulated by the vanilla exposures were all up-regulated (fold-change range: 1.6 to 3.8), whereas 3 were up-regulated (fold-change range: 2.8 to 4.6) and 3 were down-regulated (fold-change range: −1.5 to −3.2) following the strawberry exposure (Figure 4). Thus, at the molecular levels, vanilla-flavored e-cig aerosol exposure mainly activated pro-inflammatory pathways, with the up-regulation of *NF-κβ1*, *TNF-α*, *IL-6*, *IL-8*, *IL-10* and *MMP12* (Figure 4). As shown by others, ortho-vanillin is a potent inducer of IL-8 in BEAS-2B cells [48]. Together, this denotes pro-inflammatory characteristics of e-cig aerosols containing vanillin flavoring and support the notion that vanillin, as an aldehyde, is a potent inducer of transcriptional damage [50].

In summary, several *in vitro* studies seem to point in the direction that the cytotoxicity of e-liquid is dependent on the presence and number of flavoring chemicals found in e-liquids rather than solely on the presence of nicotine [20,48,62,63,64]. Our data are in line with this concept as our ALI experimental model using BEAS-2B cells was able to differentiate between the effects of two e-cig aerosols containing different flavors, namely strawberry, composed of a complex mixture of flavoring chemicals, and vanilla, composed of a simpler flavoring chemical matrix (Figure 4). Our ALI *in vitro* model enabled us to highlight that the toxicity related to the strawberry-flavored ENDS aerosol was mostly seen through alterations in redox-oxidative stress levels, whereas that of the vanilla-flavored ENDS aerosol was mainly observed at the molecular level with the dysregulation of genes involved in biotransformation, inflammation, and oxidative stress processes (Figure 4). 

## 5. Conclusions

Overall, specific properties of the ENDS aerosols, including: nicotine formulation and content, PG/G ratios, number and concentration of flavoring chemicals, and carbonyl levels, could all contribute to the ENDS aerosol toxicity [65]. This highlights the regulatory implications of the findings of this study, as flavoring chemicals in e-liquids could be regulated by governmental agencies. Flavoring chemicals that are heated and aerosolized through an ENDS device and that are documented as being highly toxic by inhalation in *in vitro* and/or in vivo, including in utero models, could be forbidden of use in e-liquid products. Thus, this study provides additional toxicological information which could be used to regulate flavoring chemicals in ENDS products to better protect ENDS users of all ages. Since e-liquid flavors are appealing and attract youth and young adult ENDS users [66], more research on the pulmonary effects of heated and aerosolized flavoring chemicals are urgently needed.

## Figures and Tables

**Table 1:** Designed primers used for human genes.

Genes	Gene Primers (5′–3′)
ACTB	Forward–GGACCTGACTGACTACCTCAT Reverse–CGTAGCACAGCTTCTCCTTAAT
ALDH-3A1	Forward–TGTTCTCCAGCAACGACAAGG Reverse–AGGGCAGAGAGTGCAAGGT
CYP-1A1	Forward–TCGGCCACGGAGTTTCTTC Reverse–GGTCAGCATGTGCCCAATCA
HPRT	Forward–CGAGATGTGATGAAGGAGATGG Reverse–TTGATGTAATCCAGCAGGTCAG
IL-6	Forward–ACTCACCTCTTCAGAACGAATTG Reverse–CCATCTTTGGAAGGTTCAGGTTG
IL-8	Forward–ACTGAGAGTGATTGAGAGTGGAC Reverse– AACCCTCTGCACCCAGTTTTC
MMP-9	Forward–TGTACCGCTATGGTTACACTCG Reverse–GGCAGGGACAGTTGCTTCT
MMP-12	Forward–GCATGGGCTAGGATTCCACC Reverse–CATGAACCGTGAGGATGTTGA
NQO-1	Forward–GGATACTGAAAGTTCGCAGGG Reverse–GAAGAGCACTGATCGTACTGGC
SOD-1	Forward–GGTGGGCCAAAGGATGAAGAG Reverse–CCACAAGCCAAACGACTTCC

**Table 2:** Nicotine, propylene glycol and glycerol concentrations found in various fourth-generation ENDS aerosols.

Device Type & Flavor	E-Liquid Nicotine Concentration	ENDS Aerosol–Nicotine (mg/puff)	ENDS Aerosol–Propylene Glycol (PG) (mg/puff)	ENDS Aerosol–Glycerin (G) (mg/puff)
JUUL menthol	3% nicotine salt	0.0735	2.13	0.614
JUUL menthol	5% nicotine salt	0.0987	1.55	0.515
JUUL Virginia tobacco	5% nicotine salt	0.112	1.66	0.544
JUUL Mango	5% nicotine salt	0.145	2.09	0.639
Puff Bar Cool mint	5% nicotine salt	0.378	4.05	3.26
Puff Bar Café latte	5% nicotine salt	0.148	1.45	1.19
Puff Bar OMG	5% nicotine salt	0.358	3.76	2.56
Posh Vape Crème brûlée	6% nicotine salt	0.28	4.58	3.06

## Data Availability

Data are available upon reasonable request.
